# COVID-19 in B Cell-Depleted Patients After Rituximab: A Diagnostic and Therapeutic Challenge

**DOI:** 10.3389/fimmu.2021.763412

**Published:** 2021-11-03

**Authors:** Anna Furlan, Gabriella Forner, Ludovica Cipriani, Elisa Vian, Roberto Rigoli, Filippo Gherlinzoni, Piergiorgio Scotton

**Affiliations:** ^1^Hematology Unit, Azienda ULSS2 Marca Trevigiana, Treviso, Italy; ^2^Infectious Disease Unit, Azienda ULSS2 Marca Trevigiana, Treviso, Italy; ^3^Microbiology Unit, Azienda ULSS2 Marca Trevigiana, Treviso, Italy

**Keywords:** COVID-19, SARS-CoV-2, rituximab, anti-CD20 therapy, B cell-depletion, B cell immunodeficiency, hyperimmune convalescent plasma, anti-SARS-CoV-2 monoclonal antibodies

## Abstract

B cell-targeting strategies such as rituximab are widely used in B cell hematologic malignancies, rheumatologic and musculoskeletal diseases and a variety of autoimmune disorders. The purpose of this paper is to illustrate how exposure to anti-CD20 treatment profoundly affects B cell functions involved in anti-SARS-CoV-2 immunity and significantly impacts on the clinical and serological course of SARS-CoV-2 infection, long term immunity and vaccine responses. The data presented here suggest that the effects of B cell-depleting agents on adaptive immunity should be taken into account for the proper selection and interpretation of SARS-CoV-2 diagnostics and to guide appropriate therapeutic approaches and protective measures. Combination therapeutic strategies including immunotherapy in association with prolonged antiviral treatment may play a decisive role in the setting of B cell immune deficiencies.

## Introduction

Anti-CD20 antibody (aCD20)-based B cell-depleting strategies such as rituximab are widely used in B cell hematologic malignancies (1) and across a variety of autoimmune disorders (2).

Upon antigen exposure, B cells have the ability to form memory cells or differentiate into plasmablasts and plasma cells (3). Memory B cells (MBCs) are central to long term immunity, since they preserve the epitope memory and give rise to new antibody secreting cells with improved specificity upon repeated antigen exposure. In addition to their roles as precursors to antibody-secreting cells, B cells can function as professional antigen presenting cells, especially in the context of cognate interactions with T cells that recognize the same antigenic target (4).

Treatment with rituximab results in complete B cell-depletion within 72 h. Recovery of B cell counts usually starts only 6-9 months after the completion of therapy, and normal levels are obtained after 9-12 months (5). While plasma cells and existing antibody levels are not affected, depletion of B cells following rituximab decreases humoral immune responses to primary antigens; in addition, as a result of prolonged decrease in MBCs, antibody production to recall antigens is decreased even 6-10 months after treatment (6).

The antibody response to SARS-CoV-2 infection has been shown to be directed to multiple targets of the virus including different epitopes of the spike protein, with those that target the receptor-binding domain (RBD) considered neutralizing. Other antibodies target the nucleocapsid protein NCP or non-structural proteins (7). Neutralizing antibodies are detectable within 7 to 15 days of disease onset, and levels increase until days 14-22, before plateauing and then decreasing.

The vast majority of subjects recovering from SARS-CoV-2 infection develop T cell and B cell memory. Remarkably, although the SARS-CoV-2 IgG antibody response begins declining beyond 20 days post-symptom onset, MBCs increase in number and affinity in the 6 months post-infection, implying an ongoing immune response despite the apparent clearance of the virus (8).

Thus, a decline in serum antibodies over time in convalescence may not reflect waning of immunity, since it appears very likely that persistent MBCs will rapidly give rise to plasma cells secreting antibodies with improved specificity on re-exposure.

Although antibody response is a central component of vaccine efficacy, memory B cells may also be important for long-term protection and the ability to respond to emerging variant strains.

In SARS-CoV-2 naive subjects, MBCs specific for full-length spike protein and RBD, as well as an optimal neutralizing antibody response are efficiently primed by mRNA vaccination after the second dose.

In SARS-CoV-2 recovered individuals, conversely, a significant boosting of antibody and MBC responses has been observed after the first vaccine dose, with no further increase after the second dose (9). The response strongly correlates with levels of pre-existing MBCs, identifying a key role for MBCs in mounting recall responses to SARS-CoV-2 antigens.

Definitive assessment of SARS-CoV-2 long term immunity following infection or vaccination will require prolonged follow up, measuring not only specific antibodies but also cellular responses and correlating these with re-infection.

The purpose of this paper is to illustrate how exposure to aCD20 treatment profoundly affects B cell functions involved in anti-SARS-CoV-2 immunity and significantly impacts on the clinical and serological course of SARS-CoV-2 infection, long term immunity and vaccination responses. Impairment of humoral immune responses bears upon the sensitivity of diagnostic tools and the efficacy of the treatment options currently available for COVID-19. Patients treated with aCD20 have not been specifically addressed in the clinical trials evaluating antiviral drugs and immunotherapy, and might benefit from combination approaches where passive immunization plays a decisive role in the absence of an appropriate antibody response.

## Pitfalls in SARS-CoV-2 Diagnostics

A number of reports on both lymphoma and rheumatic diseases (RDs) patients with SARS-CoV-2 infection after rituximab therapy, described a prolonged clinical course characterized by transient clinical improvement following standard steroid and/or remdesivir treatment (10–13) or tocilizumab (12, 13), and subsequent early relapse or exacerbation of the clinical and radiological picture.

Clinical relapse occurred in some cases even despite early negativization of PCR in nasopharingeal swab (NS) (10, 13, 14). Interestingly, 8-20 days after the first negative result on NS, viral replication was detected only in low respiratory tract samples (bronchoalveolar lavage (BAL) fluid or sputum) in 3 of 4 non-Hodgkin lymphoma (NHL) patients reported by our group (10), in line with previous NHL case reports (13, 14).

aCD20-related impairment of primary and secondary humoral responses may increase the risk of early relapse of symptoms after apparent clinical and/or microbiological recovery as well as the risk of reinfection. This suggests low threshold for repeat testing, including high-sensitivity methods to allow timely treatment and guide protection measures even shortly after or in the presence of negative results.

Viral load in the low respiratory tract may remain higher for longer periods and decrease more slowly compared with nasopharyngeal load. Thus, bronchoscopy with BAL represents a significantly more sensitive test in case of persistent clinical suspicion, especially in immunocompromised patients where viral clearance is delayed.

SARS-CoV-2 plasma viremia has been demonstrated to predict adverse outcomes in terms of severe disease and mortality (15–19).

In studies on both hospitalized and non-hospitalized patients from the general population with confirmed SARS-CoV-2 infection on NS, the reported proportion of individuals with SARS-CoV-2 viremia ranged from 10.4% to 32.9% (15–19). RNAemia was detected more frequently in individuals requiring hospitalization and who developed severe disease including ICU admission and mechanical ventilation (15). Moreover, it was closely associated with increased markers of inflammation, such as C-reactive protein and IL-6 levels (17, 18) and organ damage (19).

In patients with detectable plasma viremia, the median baseline viral load was 2.4 to 2.7 log RNA copies/mL (15, 18), which was significantly lower than that detected in respiratory samples.

Interestingly, median NP Ct has been shown to be lower, reflecting a higher viral load, in patients with detectable plasma RNA (27.1) than in patients with non-detectable plasma RNA (31.6) (16). As previously observed, however, SARS-CoV-2 RNA can be undetectable in NS of B cell-depleted patients in spite of positive viremia, due to the low sensitivity of PCR in upper respiratory samples in this setting.

A previous report of an immunocompromised allo-transplanted acute myeloid leukemia patient (20) showed that SARS-CoV-2 viremia inversely correlated with anti-SARS-CoV2 antibody production and that antibody detection coincides with viral clearance in plasma. Moreover, viremia could be detected in an early phase of infection, before the onset of symptoms and detection of SARS-CoV-2 by PCR in NS.

Moreover, in B cell-depleted individuals, viral replication assessed by PCR in NS was found to be persistently positive in a proportion of patients notwithstanding the clearance of circulating SARS-CoV-2 and resolution of symptoms after effective treatment (21). In the above mentioned study Ct values in clinically recovered patients with persistent PCR positivity in NS were reported to be <30, reflecting more likely true virus replication rather than residual viral RNA. Although there is no consensus, a Ct ≥29, corresponding to around <1000 RNA copies/mL, has been identified by some authors as a threshold level above which culture positivity rate and transmission risk are low (22, 23).

Thus, negativization of viremia may represent an early predictor of clinical response and favorable outcome in spite of delayed PCR negativization in respiratory samples. Whether, on the other hand, detection of viremia in immunocompromised B cell-depleted individuals represents a predictor of adverse outcomes similar to the general population, remains to be defined.

Immunocompromised patients are underrepresented in the studies assessing viremia and the prevalence and quantitation of plasma RNA as well as its prognostic significance in the population of B cell-depleted patients has not specifically been assessed. Data on detection of viremia in case reports and small case series are reported in **Table 1**.

**Table 1:** Reports on immunotherapy strategies in B cell-depleted patients after aCD20 therapy.

	Hueso et al. (21)	Furlan et al. (10)	Ormazabal et al. (12)	Martinot et al. (11)	Malsy et al. (13)	Rabascall et al. (24)	Kos et al. (14)
**Patient characteristics, baseline conditions and treatments**							
N patients	17 B cell-depleted (15/17 treated with aCD20)	4	2	1	1	1	1
Baseline conditions	B NHL, multiple sclerosis common Variable immune deficiency	B NHL	B NHL	B CLL	B NHL	Myasthenia gravis	B NHL
Disease status	CR 11/17; PR 3/17; PD2/17; Not attributed 1/17	CR	CR	CR	CR	Symptom control	CR
B cell-depleting agent	R-chemotherapy Rituximab/obinutuzumab maintenance	R-chemotherapy Rituximab maintenance	R-chemotherapy Rituximab maintainance	R-chemotherapy	O-chemotherapy Obinutuzumab maintenance	Rituximab (2 times per year)	R-chemotherapy Rituximab maintenance
**Laboratory values at verification of COVID-19**							
Absolute lymphocyte count, median (range),/mm3	NR	469.22 (370.0-943.36)	400.0 (Pt 1), 200.0 (Pt 2)	70.0	480.0	NR	931.0
B cell count	Undetectable in all patients treated with aCD20 (15/17)	Undetectable	Undetectable	Undetectable	Undetectable	NR	Undetectable
Gammaglobuli nemia	Hypogammaglobulinemi a in 15/17 (median 3.5, range 1.8-14 g/L) 2/17 received Ig replacement	Normal IgG (3/4)Low IgG (1/ 4)	Low-normal IgG (last dose of Ig replacement two weeks before) (Pt 1), low IgG (Pt 2)	Hypogammaglobulinemia (1.2 g/L)	Low-normal IgG	NR	Low IgG
**SARS-CoV-2 PCR and specific IgG response**							
SARS-CoV-2 anti-spike IgG (U/mL)	Negative in all patients	Negative in all patients assessed (3 of 4)	Negative	Negative	Negative	Negative	Negative
SARS-CoV-2 PCR on NS	NR	Positive at onset in all patients Negative at relapse in 3/4 (thereafter positive on sputum/BAL)	Positive at onset	Positive at onset	Positive at onset. Negative at First and subsequent relapses (thereafter positive on sputum)	Positive at onset	Negative at onset and through the whole course (positive on BAL)
Pre-treatment PCR Ct values on respiratory samples, median (range)	Median NE (range 16- 39)	28.5 (22-32)	23.0 (Pt 1), 28.25 (Pt 2)	NR	34	NR	NR
SARS-CoV-2 viremia	Positive in 9/17 (assessed prior to CP) Median NE (range 1.4-4.3 log RNA copies/mL)	NA at onset. Positive in 1/4 at relapse PCR Ct: 32	NA	Positive (assessed prior to CP) 3.94 log RNA copies/mL	NA at onset. Positive at second and subsequent relapses Viral load NR	NA	NA
**Clinical course, anti-COVID-19 treatment and outcomes**							
COVID-19 severity (WHO)	Moderate (5/17) Moderate-severe (10/17) Severe (2/17)	Moderate	Severe	Severe	Moderate	Moderate	Moderate
Resolutive COVID-19 therapy	CP	CP + 10-day remdesivir	CP (300 ml x 1 dose (Pt 1), 300ml x 2 doses, 28 days apart (Pt 2))	CP (200 ml x 4 consecutive days)	CP (2 courses of 6 units, each consisting of 2 units/d administered every other day)	MoAbs (Casirivimab- imdevimab)	High dose IV Ig, 25g/d x 5 consecutive days
Clinical relapses after temporary responses, before resolutive therapy, number	1 in 3/17	1 in 4/4	2 (Pt 1), 2 (Pt 2)	1	3	1	0
Previous COVID-19–specific treatments	Previous treatments administered to 11/17 patients: steroids, hydroxychloroquine, tocilizumab, remdesivir, lopinavir-ritonavir	Steroids, 5- day remdesivir	Steroids, hydroxychloroq uine, tocilizumab anakinra, 10 day-remdesivir	Steroids, IV Ig, hydroxychloroq uine, 5-day remdesivir	Steroids, 10 day- remdesivir, 5- day remdesivir	Steroids 5- day remdesivir,	None
Time from COVID-19 symptoms onset to resolutive therapy, median (range), days	56 (7-83)	NR	85 (Pt 1), 78 (Pt 2)	63	94	54	17
Time for oxygen weaning after resolutive therapy, median (range), days	5 (1-45)	NR	NR	NR	NR	Not applicable	NR
Time to defervescence after resolutive therapy, median (range), days	Within 48 h	1.5 (1-2)	NR	NR	NR	Within 24 h	NR
Time to negative PCR (NS/BAL/blood) after resolutive therapy, median (range), days	7 to 14 (blood) in 9 patients with positive viremia	10.6 (10-12)	~20 (after single dose in Pt 1 and after second dose in Pt 2)	7	21	7	NR
Total time to negative PCR, median (range), days	NA	48.5 (33-77)	~90/~120	70	~120	63	NR
Total hospitalization time, median (range), days	NA	29.5 (17-38)	NR	73	NR	5	24
Length of Hospital stay after resolutive therapy, median (range), days	7 (2-14)	NR	NR	10	NR	Not applicable	7
Death due to COVID-19	1/17	0	0	0	0	0	0

Patient characteristics, COVID-19 related clinical course and outcomes and additional laboratory and clinical data.

aCD20, anti-CD20; NHL, Non Hodgkin lymphoma; CLL, chronic lymphocytic leukemia; Pt 1, patient 1; Pt 2, patient 2, NE, not evaluable; NR, not reported; NA, not assessed; CP, convalescent plasma; Ig, immunoglobulin; MoAbs, monoclonal antibodies.

Based on the above observations, we advocate the potential role of viral detection in the blood and in the low respiratory tract as diagnostic tools in immunocompromised patients in whom delayed viral clearance is expected and molecular tests in upper respiratory samples have low sensitivity. Consistently, assessment of infection resolution should rely on high sensitivity methods. RT

As will be described later, serological results are also of limited use and should be interpreted cautiously in this setting.

## Clinical and Virological Course Of SARS-CoV-2 Infection

As a result of aCD20-related impairment of adaptive immunity, time to viral clearance appears to be significantly prolonged compared with the general population. In a series of NHL patients successfully treated with a combination of remdesivir and hyperimmune convalescent plasma (CP), time to ultimate negative PCR ranged from 33 to 77 days from the first positive result (10); cases of persistent infection lasting for a few months have also been reported (12). Ct values in patients with long-standing PCR positivity on respiratory samples assessed before effective treatment ranged between 20 and 30, consistent with actual viral replication (10, 12). A complete and sustained viral clearance assessed by PCR on blood or respiratory samples appears to be a requirement for stable clinical improvement in immunocompromised patients. Protracted PCR positivity is associated with a relapsing course, where clinical recurrences are accompanied by worsening radiological picture (10, 13, 25) possibly related to enhanced immunological activation and inflammatory response. Total hospitalization time, consistently, has been reported to be considerably longer compared with the general population (median 12 days), with durations that may extend up to a few months (13).

Complete B cell-depletion for up to 48 weeks can be observed after a single dose of rituximab (5). Accordingly, the clinical, virological and serological course, with particular reference to time to viral clearance and development of specific antibody response, does not seem to be affected by the number of rituximab doses administered (7) but rather by the duration from most recent exposure.

Although significantly prolonged, clinical course of COVID-19 has been reported to be moderate in severity, with no progression or delayed progression to severe disease or multi-organ involvement both in primary (25, 26) and acquired (10, 27) humoral immune deficiencies.

Anti-SARS-CoV-2 antibodies and immune complexes have in fact been recognized as playing a role in monocyte or alveolar macrophage activation, thereby contributing to sustained secretion of proinflammatory cytokines and the consequent development of severe pulmonary disease (28, 29).

Consistently, it has been observed in the general population that non seroconvertors may have lower levels of blood inflammatory biomarkers and lower disease severity (30).

In light of these considerations, a potential therapeutic use of rituximab has been advocated in the management of specific complications of COVID-19 such as thrombotic or inflammatory lung complications persisting beyond acute infection, in the presence of negative/low viral loads and/or seroconversion (31).

As previously stated, immunoglobulin (Ig) levels may not be significantly affected after rituximab despite long standing B cell-depletion. There does not seem to be a correlation between existing Ig levels and the duration and outcome of the infection nor with the likelihood of seroconversion (10). Accordingly, vaccine-specific antibody responses cannot be predicted on the basis of baseline Ig levels in this setting.

## Therapeutic Implications: Is a Differentiated Approach Needed?

Remdesivir is to date the only COVID-19 antiviral treatment with a reported efficacy (32). A 5-day treatment has been demonstrated to be not inferior to a 10-day course in patients with severe COVID-19 disease not requiring mechanical ventilation (33).

The administration of anti-SARS-CoV-2 convalescent plasma (CP) to hospitalized patients with COVID-19 late in the course of illness and with severe pneumonia has not been demonstrated to significantly impact on the clinical outcomes (34). Furthermore, a recently published randomized study showed that early administration of CP to outpatients at high risk of severe COVID-19 within one week after the onset of symptoms did not prevent disease progression (35).

However, the specific case of humoral immune deficiencies was not addressed in the above mentioned trials.

Yet, a number of clinical reports have suggested that passive immunization with CP might represent a valid therapeutic option in the context of protracted COVID-19 symptoms in B cell-depleted patients unable to mount a specific humoral response to SARS-CoV-2 (11, 12, 21, 25). CP has been shown to be effective after failure or partial/temporary response to steroids and/or remdesivir and to tocilizumab. In the lack of specific convalescent serum, high-dose IV Ig administration could also be beneficial (14).

Also, there is a rationale in treating patients who are unable to mount a specific antibody response with CP soon after exposure to SARS-CoV-2 in order to prevent symptomatic COVID-19. Data in this specific setting, however, are lacking.

Long standing/relapsing course of COVID-19 has been observed despite evidence of a robust T cell response in patients with both primary (26) and secondary (13, 14, 21) humoral immune deficiencies.

Specific T cell responses to SARS-CoV-2 seem, therefore, not sufficient to control the infection in the absence of neutralizing antibodies. Passive immunotherapy appears to be beneficial in B cell-depleted individuals as it provides the neutralizing SARS-CoV-2 antibodies that are mandatory for viral clearance.

On the other hand, T cells may play a central role in passive immunotherapy as they work synergistically with the antibodies brought by CP (21). Importantly, T cells recognize a wider range of epitopes outside the antibody binding sites that are susceptible to mutational escape (36).

Depletion or functional impairment of T cells, for example following treatment with disease modifying antirheumatic drugs (DMARDs) (37) may account for the failure of CP administered as a single agent in B cell-depleted individuals with RDs and explain why in this population rituximab should be considered a risk factor for unfavorable outcome with a high rate of severe COVID-19 leading to a high mortality rate (23.1%) (38). On the other hand, RDs patients without B cell deficiency treated with both synthetic and targeted synthetic DMARDs do not seem to be at increased risk of respiratory life-threatening complications from SARS-CoV-2 (39), probably owing to the inhibitory effect of treatment on inflammatory response driving SARS.

Some data (40) highlight the issue of the potential emergence of SARS-CoV-2 genomic variants with reduced susceptibility to neutralization after administration of CP in immunocompromised individuals where prolonged viral replication occurs. Caution is especially suggested in the use of CP in patients with immunosuppression of both T and B cell arms, since in such cases the antibodies administered have little support from cytotoxic T cells, thereby reducing chances of clearance and raising the potential for escape mutations. The emergence of SARS-CoV-2 mutations has also been reported in a post-rituximab B cell-deficient patient during a 5-day course of remdesivir, leading to treatment failure (11).

The above considerations support the rationale of combining CP with an extended course of an agent interfering with viral replication in the setting of severe immunosuppression.

Successful treatment with a combination of remdesivir and CP after failure of single agent remdesivir (10, 13), CP or standard IV Ig (25) in the setting of protracted disease has been reported both in post-rituximab and in primary B cell immune deficiencies.

Dramatic clinical responses have been described in terms of defervescence, respiratory recovery and radiological improvement. In line with a report of B cell-depleted patients successfully treated with CP alone (21), resolution of fever and weaning from oxygen support occurred within 24-48 h and a median of 4 days from the first CP transfusion, respectively.

Differently from previous treatments (steroids +/- standard 5-day course of remdesivir), the combination of CP with a 10-day course of remdesivir resulted in sustained responses with no signs of virological nor clinical relapse after a median follow up of 6 months (10).

In the face of the rapidity of clinical response, time to viral clearance after passive immunotherapy is highly variable (range 7 to 112 days) (12, 21). In a small series of NHL patients treated with CP in association with an extended course of remdesivir, SARS-CoV-2 RNA in NS or BAL samples turned negative after a median of only 10.6 days (10 to 12) (10).

As outlined before, clinical recovery often precedes PCR negativization in respiratory samples and appears to correlate more strongly with the clearance of SARS-CoV-2 viremia (21). Circulating SARS-CoV-2 RNA represents, therefore, an earlier and more sensitive indicator of virological response after passive immunotherapy in the clinical setting described here.

In addition, it should be considered that baseline medical conditions (hematological malignancies, RDs, autoimmune disorders), disease activity, concomitant/previous treatments (chemotherapeutic agents, DMARDs) that influence the T cell subset distribution and function may have a prominent role in affecting response to immunotherapy +/- antiviral agents in terms of time to clinical recovery and viral clearance.

Passive immunotherapy with anti-SARS-CoV-2 monoclonal antibodies (MoAbs) represents another therapeutic option that has been demonstrated to be most effective early in the disease process.

In patients with mild to moderate disease who are at high risk for progressing to severe COVID-19 as in the case of primary or secondary immune deficiencies, the administration of anti-SARS-CoV-2 MoAbs can be considered even >10 days after onset of symptoms in case of prolonged PCR positivity and in the absence of a detectable specific antibody response.

Moreover, recent data support the use of the MoAbs combination of casirivimab/imdevimab in hospitalized patients with COVID-19 including patients with oxygen requirement who are seronegative (41). The combination of two neutralizing antibodies (casirivimab/imdevimab) binding to different parts of the virus spike decreases the risk of losing neutralization potency against new emerging variants (42), which is of the utmost importance in patients who are unable to mount a neutralizing antibody response and in whom prolonged viral replication is expected. In this perspective, the combination of MoAbs and antiviral agents is of potential interest. Casirivimab/imdevimab has been successfully used after failure of remdesivir in a patient with mysthenia gravis on rituximab who had persistently positive PCR and negative anti-SARS-CoV-2 antibodies after 2 months of symptom onset. MoAbs treatment resulted in clinical improvement after 24 hours and PCR negativization one week following the infusion (24). Clinical studies addressing the use of MoAbs in the subset of B cell-depleted individuals with prolonged symptoms are needed.

**Figure 1:**
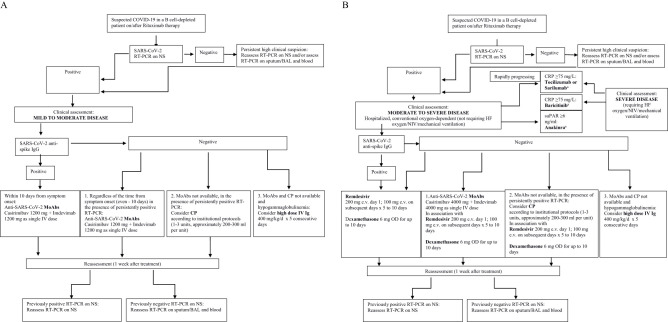
**(A)** A potential therapeutic approach to immune compromised B cell-depleted patients with COVID-19 on/after Rituximab therapy. Mild to moderate disease. MoAbs, monoclonal antibodies; CP, convalescent plasma; Ig, Immunoglobulins. **(B)** A potential therapeutic approach to immunocompromised B cell-depleted patients with COVID-19 on/after Rituximab therapy. Moderate to severe disease. MoAbs, monoclonal antibodies CP, convalescent plasmas; Ig, Immunoglobulins; HF, high flow; suPAR, plasma Soluble Urokinase-Type Plasminogen Activator Receptor. ^a^The role of anti-inflammatory drugs in B cell-depleted patients with COVID-19 on/after Rituximab may be questionable due to the lower levels of inflammatory biomarkers typically observed in non seroconverters and needs to be assessed in dedicated studies.

In conclusion, passive immunotherapy with both convalescent plasma and MoAbs may represent a key therapeutic tool in treating patients unable to mount an appropriate humoral response (**Figure 1**). However, these findings are based on case reports and small series and, as such, should be taken cautiously as more rigorous research including randomization and a larger sample size is needed.

## Impact of B Cell-Depletion on Serological Response to Primary SARS-CoV-2 Infection and Vaccine Response

A common finding in individuals treated with B cell-targeted agents is failure to seroconvert after primary infection, independent of the baseline medical conditions and concomitant/previous treatments, as well as independent of viral load and time to viral clearance (10, 12, 14).

aCD20 therapy is expected to impair not only the production of protective antibody by effector B cells, but also the development of virus-specific memory B cells which are responsible for faster antibody production upon re-exposure to antigen.

Following SARS-CoV-2 infection a majority of individuals develop a strong CD4+ and CD8+ T response, and some have a memory phenotype, potentially responsible for longer-term immunity (29). As discussed before, specific T-cell responses to SARS-CoV-2 seem, however, not sufficient to control primary infection in the absence of neutralizing antibodies. Longer follow up data are needed to clarify whether memory T cells formed upon primary SARS-Cov-2 infection are able to exert a protective effect upon re-exposure in the absence of a B cell response.

Interestingly, between immunocompetent individuals, non seroconverters have been demonstrated to have significantly higher cycle threshold (CT) values of RT-PCR (38.0 vs 28.0) and shorter time to viral clearance (3.0 vs 41.0 days), suggesting that low SARS-CoV-2 viral load might be insufficient to stimulate adaptive humoral immunity and generate the antibody response (30). Patients with severe humoral deficiency fail to seroconvert in spite of pre-treatment CT values lower than the general population (median 26.5, range 25.0-28.0) (10).

aCD20 treatment has been proved to significantly impair Spike and RBD (Receptor Binding Domain) specific antibody and memory B cell responses to SARS-CoV-2 mRNA vaccination (43, 44). A detectable antibody response 2-8 weeks after the second dose of mRNA vaccine has been reported in only ∼1/3 of multiple sclerosis patients on B cell-depleting therapies (43, 45).

B cell reconstitution, even at weak levels (46–48) and, consistently, longer duration from most recent rituximab exposure (47) are associated with higher rates of serological response to vaccine. Yet, in a study of chronic lymphocytic leukemia patients, less than half the subjects who received rituximab more than 12 months prior to vaccination had a detectable response (49).

In contrast to humoral response, antigen-specific CD4+ and CD8+ T cell responses to vaccination are not affected by aCD20 therapy (44, 46, 48). The question of whether B cells are essential for SARS-Cov-2 vaccination protective effect, and whether T cell responses to vaccination and primary infection might protect against infection/re-infection or, at least, prevent severe disease (50) in B cell-depleted individuals, remains to be defined.

Concomitant T cell deficiency related to the underlying medical condition or concomitant/previous exposure to chemotherapy/immunosuppressants other than aCD20 antibody might also affect long-term immunity following both vaccination and primary infection, as well as responsiveness to passive immunotherapy approaches as previously discussed.

NCCN guidelines appropriately recommend vaccination in patients with cancer, but do not address aCD20 therapy specifically and do not comment on whether to hold anti-CD20 therapy prior to vaccination nor on the optimal timing after most recent rituximab exposure (51). Since vaccine efficacy in terms of serological response is impaired for at least 6-12 months after rituximab treatment, the opportunity of a longer-term hold of rituximab must be carefully weighed against the benefits of aCD20 treatment, especially in the settings where it confers a survival benefit, and all the more as vaccine-induced T cell responses might play a protective role nonetheless.

Further insight on the kinetics of antibody and specific memory B cell responses, and their relationship to the level of peripheral B cells needs to be gained to define the optimal timing and schedule of vaccination after aCD20 treatment.

## Conclusions

In conclusion, aCD20 treatment profoundly affects B cell functions involved in anti-SARS-CoV-2 immunity and significantly impacts on the clinical and serological course of SARS-CoV-2 infection, long term immunity and vaccine responses. The effects of adaptive immunity deficiency in patients treated with B cell-depleting agents should be taken into account for the proper selection and interpretation of SARS-CoV-2 diagnostics and to guide appropriate therapeutic approaches and protective measures. To achieve stable clinical responses and minimize the risk of emergence of SARS-CoV-2 genomic variants, this subset of patients might benefit from combination regimens, including both passive immunotherapy and prolonged antiviral treatment, that need to be defined in dedicated prospective trials.

## Author Contributions

AF wrote the manuscript. GF, LC, RR, PS, and FG contributed to critical revision. All authors contributed to the article and approved the submitted version.

## Conflict of Interest

The authors declare that the research was conducted in the absence of any commercial or financial relationships that could be construed as a potential conflict of interest.

## Publisher’s Note

All claims expressed in this article are solely those of the authors and do not necessarily represent those of their affiliated organizations, or those of the publisher, the editors and the reviewers. Any product that may be evaluated in this article, or claim that may be made by its manufacturer, is not guaranteed or endorsed by the publisher.
